# Supplementary material for: Curated and harmonised transcriptomics datasets of interstitial lung diseases
Source: Data Brief. 2025 Oct 14;63:112139. doi: 10.1016/j.dib.2025.112139 (PMC12581653; doi:10.1016/j.dib.2025.112139)
Supplement: Supplementary file 1 [file mmc1.zip › Supplementary_material/RNA-seq/fastQC reports/GSE169500/SRR14057118_fastqc.html]

SRR14057118.fastq.gz FastQC Report 

FastQC Report

Mon 5 Feb 2024  
SRR14057118.fastq.gz

## Summary

- Basic Statistics
- Per base sequence quality
- Per sequence quality scores
- Per base sequence content
- Per sequence GC content
- Per base N content
- Sequence Length Distribution
- Sequence Duplication Levels
- Overrepresented sequences
- Adapter Content

## Basic Statistics

| Measure | Value |
| --- | --- |
| Filename | SRR14057118.fastq.gz |
| File type | Conventional base calls |
| Encoding | Sanger / Illumina 1.9 |
| Total Sequences | 10693350 |
| Sequences flagged as poor quality | 0 |
| Sequence length | 25-373 |
| %GC | 49 |

## Per base sequence quality

## Per sequence quality scores

## Per base sequence content

## Per sequence GC content

## Per base N content

## Sequence Length Distribution

## Sequence Duplication Levels

## Overrepresented sequences

| Sequence | Count | Percentage | Possible Source |
| --- | --- | --- | --- |
| CAATATCAAGGAAGGGCAGGCGTGATGGCTTATTTGTTTTGTATTCAATG | 109342 | 1.0225233439474064 | No Hit |
| TTCTTGTAGATGGCTGCTCTAAAAAGACAAATGAATGGGGAAAGACAATC | 97215 | 0.9091164134719242 | No Hit |
| ACCAATGTCATAGGGTGCAATATCTACAATAGGTAGTCTCACAGCCTTGC | 95574 | 0.8937704274151692 | No Hit |
| AGAGCATGACCGATGGATTCCAGTTCGAGTATGGCGGCCAGGGCTCCGAC | 90018 | 0.8418129024113118 | No Hit |
| GGGAATGTCTACGTGCGTATGCACGTGGCACTCTCTGCCCGAGGTCCGGG | 83079 | 0.7769221057947229 | No Hit |
| GGGCCACGAGCTGAGTGCGTCCTGTCACTCCACTCCCATGTCCCTTGGGA | 80050 | 0.7485960900933758 | No Hit |
| TGGTTGCACGAAACACACTGGGGAATGGAGCAAAACAGTCTTTGAATATC | 77935 | 0.7288174426162054 | No Hit |
| AAGCTCAGGGAGAGCCCTGTTAGGGCCGCCTCTGGCCCTAGTCTCAGACC | 74497 | 0.6966666199086348 | No Hit |
| ACACTGAGGACTCTGTTCCTCTCCTTTCCGCCTAGGGGAAAGTCCCCGGA | 59170 | 0.5533345490421617 | No Hit |
| GCTGCTACAGGAGAATAGCAGACAGGTATAGTTAAGAAACAACTTTATTT | 59095 | 0.5526331785642479 | No Hit |
| ATGTTCTGGGAGGCCTCGGTGGACATCAGGCGCAGGAAGGTCAGCTGGAT | 57702 | 0.5396063908877947 | No Hit |
| ACACTGAGGACTCTGTTCCTCCCCTTTCCGCCTAGGGGAAAGTCCCCGGA | 49445 | 0.4623901770726666 | No Hit |
| TTTACACCTGCTAGGGTGTTCAAAGGTCAGTGCTATAGAAATTCAGTATC | 44697 | 0.4179887500175342 | No Hit |
| CTAAGTGCTGTGTTGTCGTTCCCCCTGCTTAAAATAAAGTTGTTTCTTAA | 40111 | 0.37510228319469574 | No Hit |
| TCTGCAATGCAATCACAATGCCCAAACTAGACCTGCCATTTCTCACACAC | 32689 | 0.3056946607003418 | No Hit |
| ATGTTCTGGGAGCCTCGGTGGACATCAGGCGCAGGAAGGTCAGCTGGATG | 32604 | 0.3048997741587061 | No Hit |
| AAAGCCAAGAAAACCAACGATGCCAGATACTGAATTTCTATAGCACTGAC | 31713 | 0.29656749288108963 | No Hit |
| ACACCCACCGCAACTGTCTGTCTCATATCACGAACAGCAAAGCGACCCAA | 26217 | 0.24517106425956317 | No Hit |
| TGGTTCCTGGCAAGCCCATGTGTGTTGAGAGCTTCTCAGACTATCCACCT | 24235 | 0.22663618042989336 | No Hit |
| ACTTGATCCCAACTCATCTCTCATTTATTTCGGCTTCTTTTATTCCAGGA | 23524 | 0.2199871882992701 | No Hit |
| ATTCTTCCTACCCATGAGCATGGAATGTTCTTCCATTTGTTTGTATCCTC | 23498 | 0.2197440465335933 | No Hit |
| AGCGGTTCCGCTGCCCTGAGGCACTCTTCCAGCCTTCCTTCCTGGGCATG | 22416 | 0.20962560843888958 | No Hit |
| TGTTGTGCTAAACGCTGGGAGGGTACAAGTTGGTCATTCCTAAATCTGTG | 21933 | 0.20510878256112444 | No Hit |
| AGTGTGGCGATTCCTCAGGGATCTAGAACTAGAAATACCATTGACCCAGC | 21090 | 0.19722537838937285 | No Hit |
| TCACACTTCATGATGGAGTTGAAGGTAGTTTCGTGGATGCCACAGGACTC | 20824 | 0.19473785109437172 | No Hit |
| GAAGACGTACCTTTGTTGGAAGACATACCTTTGTTGGAAGACGTACCTTT | 18294 | 0.17107828697274474 | No Hit |
| GGTTATAATCATTGGCAATGTTGTAATCAACAACCCATGTTATTTCTACA | 18265 | 0.1708070903879514 | No Hit |
| AATAAAAGCGAAAAGAAATGAAAATGTTACACTACATTAATCCTGGAATA | 17998 | 0.1683102114865781 | No Hit |
| TTCTTGTAGATGGCTGCTCTAAAAGACAAATGAATGGGGAAAGACAATCA | 17589 | 0.16448540448035462 | No Hit |
| CAGAAGCTCTTTATGTCAAACTTCTCAAGTTGATTGACCTCCAGGTAATA | 17292 | 0.16170797738781578 | No Hit |
| TGACTGGTGATGCTGATATTGGAGTGCATCTGAGGCTGTTGTAGAAATAA | 17172 | 0.16058578462315365 | No Hit |
| CAGCATGATTTATAGTCCTTTGGGTATATACCCAGTAATGGGATGGCTGG | 16217 | 0.15165500053771738 | No Hit |
| TCAACGTACTCCATGCCTGGCAGCTTCATCCCGTACTTGTCCACGCACCA | 15241 | 0.1425278327184652 | No Hit |
| AAGAGAAAGCAGTGCAAACCTTCCCGTGGCCGCAAGCGTGGCATCTGCTG | 15127 | 0.14146174959203617 | No Hit |
| TACATCAAAGATTACATGAAATCAATCAAAGGGAAACTTGAAGAACAGAG | 14764 | 0.13806711647893316 | No Hit |
| ACCCGCAGCTTCTGCTTCTCAGTCAGAAGGTTGTTGTCCTCATCCCTCTC | 14533 | 0.13590689540695852 | No Hit |
| AACATCTGTGTTGGGGAGAGTGGAGACAGACTGACGCGAGCAGCCAAGGT | 14404 | 0.13470053818494673 | No Hit |
| ATCAAATTAATGTCTTCCAGCCTACTTGTGTCTTCCAACAAAGGTACGTC | 14393 | 0.1345976705148527 | No Hit |
| TGCGAAAAGTTACTTTCAATGGACTGAACCAGATGATTGTCATAGAACTG | 14371 | 0.1343919351746646 | No Hit |
| CGGTTGTACTTGCGGATGTAGTGCAGATAGTCTCGGCGGATGACAATGGT | 14339 | 0.13409268377075473 | No Hit |
| CACTTGGCTTCACCCGTGTGACTTTCGTAACGGGGAGAGAGAGAAAAGAT | 13785 | 0.12891189384056445 | No Hit |
| TGTCCTATAACAAGCTTAAAAACATACCAACTGTCAATGAAAACCTTGAA | 13225 | 0.1236749942721411 | No Hit |
| AGAGGCCACAGTAAGGCCCATCCGAGGTCCTGAGGAGATCTTTTCTCTCT | 13089 | 0.12240317580552398 | No Hit |
| TCTTCAAGGAGAACTACAAACCACTGCTCAAGGAAATAAAAGAGGATACA | 12881 | 0.12045804168010961 | No Hit |
| ATTCGAGGGCGGATCCTCTCTGGCGTGGTGACCAAGATGAAGATGCAGAG | 12685 | 0.11862512683116141 | No Hit |
| TGTTCCCTCTCCTCATGAGATTGGTGAAGAAAGTATTTGGCAAAGTTCTT | 12378 | 0.11575418367490076 | No Hit |
| TTCCCCCTGCGCATGCGGGACTGGCTCAAGAACGTCCTGGTCACCCTGTA | 12352 | 0.11551104190922397 | No Hit |
| TGTCCATGTCTTACTACTTTGACCGCGATGATGTGGCTTTGAAGAACTTT | 12313 | 0.11514632926070875 | No Hit |
| TGGCTCTCCCAGAGGCAGAAGGACAATGGCTGTTTCAGGAGCTCTGGGTC | 12195 | 0.11404283970879098 | No Hit |
| TTTTTGTTGCCTGTAAAGTGCTCTGGCATTGCCTGAGGATAGATGAGAAA | 11932 | 0.11158336723290643 | No Hit |
| TCTTCCAGCCTACTTGTGTCTTCCAACAAAGGTACGTCTTCCAACAAAGG | 11015 | 0.10300794418961316 | No Hit |
| ACAGTGTATCTAGCTTTGGAAAACACAGGGGTCTGCCCTGTGAGCTGCTC | 10835 | 0.10132465504261993 | No Hit |

## Adapter Content

Produced by FastQC (version 0.11.7)
